# Editorial: Open when, why, to whom? Changing challenges, perspectives and practices in a new research culture

**DOI:** 10.3389/frma.2023.1303941

**Published:** 2023-10-17

**Authors:** Rogério Mugnaini, Chris Fradkin, Shalini Urs

**Affiliations:** ^1^School of Communication and Arts, University of São Paulo, São Paulo, Brazil; ^2^MYRA School of Business, Yelawala, Karnataka, India

**Keywords:** open science, electronic publishing, scientific rigor, reproducibility, preprints, integrity

The future is open science. Transparency, scrutiny, critique, and reproducibility are slowly and steadily becoming the normative principles of science. The COVID-19 crisis added steam to this new movement, which has redefined our role, as authors, researchers, and publishers. Two decades ago, the consumers of scientific publishing were peers and colleagues of the authors. With open access, the consumers now include a diverse array of people from a diverse array of backgrounds. “Transparency” and “reproducibility” are more than buzzwords of the day; they have raised the standards for scientific rigor. Metadata fields are expanding, almost every day, to the delight of information scientists. With preprints, data are available worldwide, independent of publication schedules. Communication platforms (e.g., Zoom and Google Meet) facilitate collaboration worldwide. The scientific landscape is changing.

Articles in this Research Topic address changes and challenges in the new research culture and offer suggestions for increased accountability. Hoffberg et al. share the findings of a study on visual abstracts. Using Twitter Analytics, the authors found that visual abstracts received a significantly higher number of impressions, retweets, and link clicks than their text abstract counterparts. The findings suggest that visual abstracts increase both the awareness and readership of journal publications.

Using data from the Italian Ministry of Health, Pozzo and Virgili examined the emergency readiness of local administrations in the inner areas of Italy, amid the COVID-19 pandemic. The authors contend that many administrations were underequipped with the management infrastructure required to comply with “social distancing precautions and to be effective with positive case tracking” (Pozzo and Virgili, p. 3). The authors voice the concern that Italy's handling of the pandemic was not consistent with their commitment to the 17 United Nations Sustainable Development Goals (United Nations, 2015).

The Registered Report System was designed to reduce questionable research practices and bolster reproducibility in psychology studies (Nosek et al., 2018). In an opinion piece based on the first author's real-life experience, Sasaki and Yamada question the adaptability of such a system. The authors relay that they had a protocol manuscript accepted by a journal under the condition that they deliver the full manuscript 2 months later. As the in-principle acceptance was early in the COVID-19 pandemic, the authors were unable to conduct any laboratory experiments, and were, thus unable to meet the 2-month deadline. The authors requested a post-pandemic extension on the deadline. The journal denied their request and thereby deprived the authors of an accepted publication. This highlights the need for flexibility in protocol, within well-intentioned open science measures.

Fradkin and Mugnaini propose open science indicators (open data, open material, and preregistration) as article-specific metadata fields. The authors base their case on the inclusion of funding disclosures as metadata fields and cite its impact on the scientometric landscape. They contend that the inclusion of open science indicators as metadata fields may have an equally transformative effect on the scientific publishing community.

Turki et al. discuss the importance of Digital Object Identifiers (DOIs) and their critical role in the accessibility and discoverability of online publications. The authors contend that journals and institutions in developing nations are at a disadvantage in terms of access to and the acquisition of DOIs. Although the authors applaud the Global Equitable Membership (GEM) program launched by *Crossref* for its efforts to address this issue, they stress the need for more initiatives in this area.

These articles remind us that open science innovations must regularly be monitored and refined. Although celebration is in order for the steps we have taken, in the recent years of electronic publishing, our responsibility is to look back on those steps and review not just the distance we have traveled but the quality of the journey and the refinements we can make for future steps. As scientists, we sometimes paint a picture of the scientometric landscape as being rife with splendid innovations, without asking ourselves, “What more could we have done?” At this point in our open science journey, we must look beyond intention and assess a work that is in progress. A journey that defines its destination.

## Author contributions

RM: Conceptualization, Writing—review and editing. CF: Conceptualization, Writing—original draft. SU: Writing—review and editing.
